# MRI‐measured tendon retraction distance is associated with EMG‐confirmed neurotrauma in proximal hamstring avulsion

**DOI:** 10.1002/jeo2.70672

**Published:** 2026-03-07

**Authors:** David Wood, Miloš Spasojevic, Sofie French, Ran Wei, Sebastian Fung, Karl Ng

**Affiliations:** ^1^ North Sydney Orthopaedic and Sports Medicine Centre Sydney New South Wales Australia; ^2^ University of Queensland St Lucia Queensland Australia; ^3^ Australian Institute of Musculoskeletal Research Sydney New South Wales Australia; ^4^ Kingston Hospital NHS Foundation Trust Kingston upon Thames UK; ^5^ St Vincent's Private Hospital Darlinghurst New South Wales Australia; ^6^ Department of Neurology and Clinical Neurophysiology Royal North Shore Hospital and The University of Sydney Sydney New South Wales Australia

**Keywords:** athlete proximal hamstring, proximal hamstring avulsion, proximal hamstring nerve injury, proximal hamstring repair, proximal hamstring sciatic nerve

## Abstract

**Purpose:**

Complete proximal hamstring avulsion injuries are anatomically complex because of their proximity to the sciatic nerve. This study characterises neurological abnormalities following injury and surgical repair. Although denervation has been described, its severity, pattern and diagnostic thresholds in relation to magnetic resonance imaging (MRI) findings, anatomical innervation and tendon retraction distance remain poorly defined.

**Methods:**

In this prospective longitudinal cohort study, 18 patients undergoing surgical repair of MRI‐confirmed complete proximal hamstring avulsion were evaluated using serial electromyography (EMG) and MRI performed preoperatively and postoperatively over 12 months. Tendon retraction distance, muscle and sciatic nerve MRI characteristics and EMG evidence of denervation were recorded. EMG findings were used to distinguish traumatic from postoperative nerve injury. Receiver operating characteristic analysis and Youden's *J*‐statistic were applied to determine a tendon retraction threshold associated with neurotrauma.

**Results:**

Among the 18 patients, 5 (28%) had preoperative nerve injuries. Of the 13 patients without denervation preoperatively, 3 (23%) experienced iatrogenic injuries postsurgery. SHORE scores and MRI did not differ significantly between normal and abnormal EMG cohorts, although neurological symptoms were numerically more frequent in the abnormal group. Increased tendon retraction was significantly associated with more severe EMG abnormalities in the hamstring muscles, with a 5‐cm threshold demonstrating good discrimination.

**Conclusion:**

Proximal hamstring avulsion injuries exhibit varying degrees of neuropathology and recovery. MRI‐measured retraction, not MRI signal changes, may predict neurotrauma. EMG is required to confirm denervation. Retraction distance over > 5 cm (nerve at risk distance, NARD) is associated with a substantially increased risk of neurotrauma, but the long‐term clinical consequences of underlying nerve injury, especially in the athlete, require further investigation.

**Level of Evidence:**

Level II, prospective longitudinal cohort study.

## Graphical abstract

MRI‐measured tendon retraction distance predicts EMG‐confirmed neurotrauma in proximal hamstring avulsion, identifying a nerve‐at‐risk distance beyond which neurological injury is more likely.

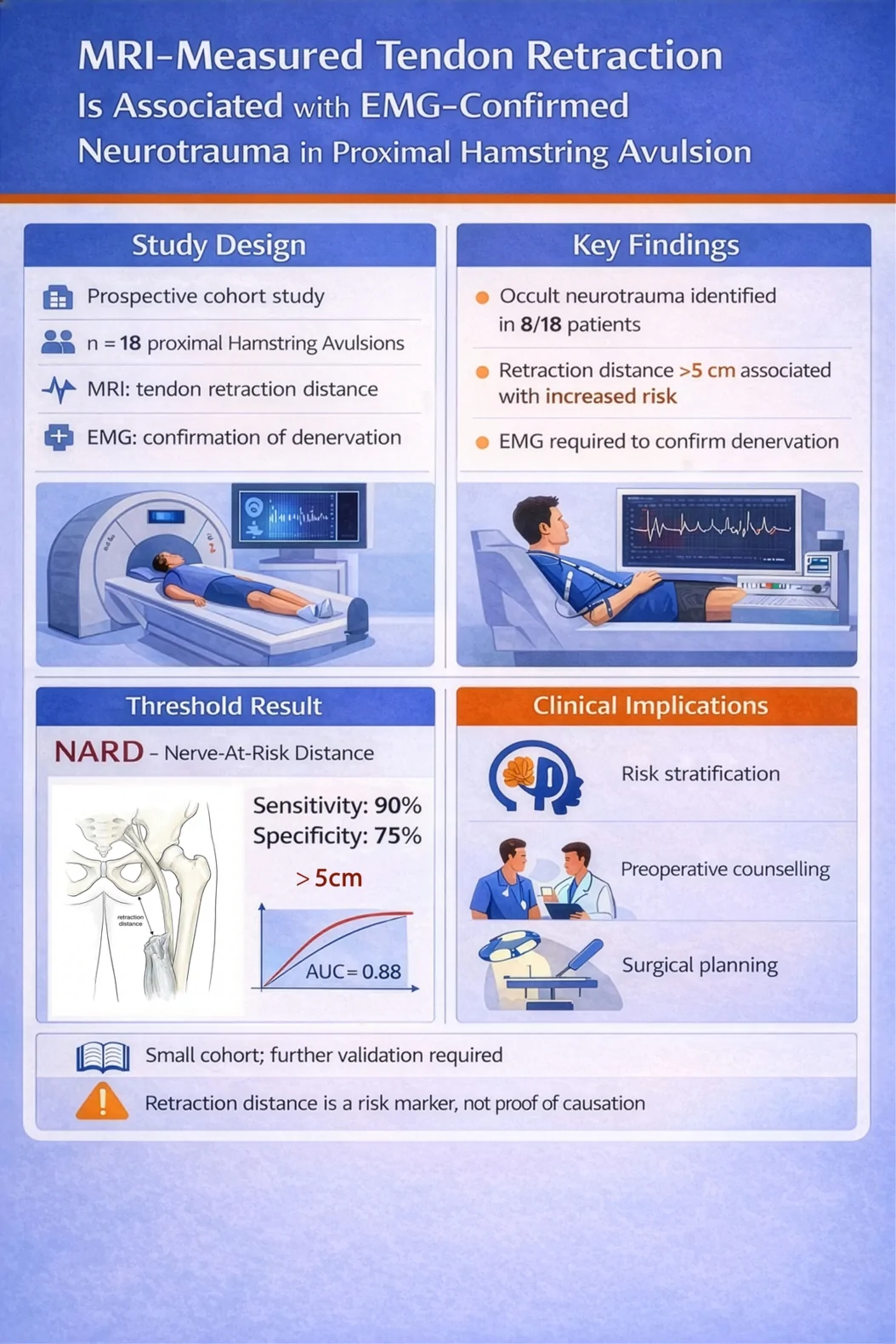

**Abbreviations**
EMG: electromyography
FPR: false positive rate
MRI: magnetic resonance imaging
MRN: magnetic resonance neurography
NARD: nerve at risk distance
NCS: nerve conduction studies
ROC AUC: receiver operating characteristics area under the curve
SHORE: Sydney hamstring origin rupture evaluation
STIR: short tau inversion recovery
TPR: true positive rate

## INTRODUCTION

Proximal hamstring avulsion injuries represent a significant athletic impairment, most commonly caused by forceful eccentric contraction of the hamstring muscles during activities such as water skiing and hurdle jumping [2, 14].

These injuries, in which the hamstring detaches from its origin at the ischium, are predominantly observed in middle‐aged individuals and demonstrate a notable gender disparity: women are more likely to sustain the injury during daily activities, whereas men sustain it predominantly in sports [14]. Accompanying sciatic nerve‐related symptoms are common and range from motor weakness to sensory disturbances and neuropathic pain [26, 27]. Nonoperative management of complete avulsions may result in persistent deficits in strength and function, although some patients manage to return to their previous levels of sporting activity [1, 11]. Surgical intervention is advocated for complete avulsions, particularly for athletes, to expedite a return to sport and enhance functional outcomes, and in sciatic nerve involvement [24, 27]. The described surgical techniques for reattaching the proximal hamstring avulsion generally yield satisfactory postoperative results in terms of strength and endurance relative to the uninjured side [28]. However, some complete avulsions can result in more substantial injury to the proximal hamstring innervation due to the close neuroanatomical relationship between the ischial tuberosity origin and sciatic nerve and its branches [19, 20]. Delayed surgical repair may increase the risk of further sciatic nerve compromise from traction and tethering, often necessitating extensive intraoperative neurolysis [15, 28].

Despite these risks, patients with sciatic nerve‐related symptoms following proximal hamstring avulsion often demonstrate improvement in motor deficits, sensory symptoms and neuropathic pain following surgical repair [27].

Current evidence does not allow reliable differentiation between traumatic and iatrogenic sciatic nerve injury, complicating prognostication and patient counselling. While magnetic resonance imaging (MRI) is the gold standard imaging modality for assessment of proximal hamstring injury itself, the optimal adjunct for evaluation of neuropathology remains undefined [12, 29].

The aims of this study were to determine whether nerve injury associated with proximal hamstring avulsion is primarily traumatic or iatrogenic, to characterise the pattern and temporal evolution of motor denervation using serial electromyography (EMG), and to assess whether MRI‐derived measures of injury severity are associated with EMG‐confirmed neural injury. It was hypothesised that more extensive avulsion injuries on MRI, including greater tendon retraction, would be associated with a higher prevalence and greater extent of denervation on EMG.

## METHODS

This is a prospective longitudinal cohort study.

### Participants

Participants, recruited by a single orthopaedic surgeon (D.W.), had MRI‐confirmed proximal hamstring avulsions and chose surgical repair, agreeing to complete up to four EMG and MRI procedures over 12 months. Ethics approval was granted by St Vincent's Hospital Human Research Ethics (2019/ETH10683). Written informed consent was provided by all participants prior to data collection.

### Variables of interest

At the preoperative consultation, the following data was collected from each participant: age, sex, side of injury, date of injury, mechanism of injury, previous treatment, injury classification [28], tendon retraction distance, sciatic nerve involvement from MR appearances, presence of preoperative neurological symptoms, injury severity, preinjury activity levels, date of surgical repair and the Sydney Hamstring Origin Rupture Evaluation (SHORE) score [9]. Any complication experienced within 12 months postoperatively was recorded for each participant.

### Timeline

Following injury, all participants underwent preoperative MRI and EMG assessment prior to surgical repair. Surgery was performed after completion of baseline imaging and neurophysiological testing. Postoperative MRI and EMG assessments were performed at 3 months, with additional assessments at 6 and 12 months in participants with abnormal findings, to distinguish traumatic from postoperative nerve injury and to evaluate recovery over time.

### Operational definitions

The mechanism of injury was classified by velocity; low (walking speed or less), medium (between walking and running speeds) or high (above running speed). All injuries were confirmed by MRI and classified according to a method previously described [28]. Tendon retraction distance was assessed on MRI. A single, trained radiologist measured tendon retraction distance, and oedema and denervation of hamstring muscle based on intensity in T1 and T2 signals. Preoperative neurological symptoms were considered present if participants reported tingling or altered sensation. Injury presentation was dichotomised into acute (< 6 weeks) or chronic. Participants' preinjury activity level was categorically classified as: recreational, competitive and elite.

### SHORE score

Functional outcomes were assessed using the SHORE score, a patient‐reported outcome measure evaluating pain, walking/running ability, activity limitation, sitting/driving tolerance, stair negotiation and symptoms of ache and tightness, assessed both preinjury and at follow‐up.

Participants' pain and functional outcome was assessed at their preoperative, 6‐ and 12‐month appointments. The patient‐reported outcome measure was devised and validated by the senior author (D.W.) [9]. (Table 1, SHORE score).

**Table 1:** SHORE score.

Before injury/current	SHORE—Sydney hamstring origin rupture evaluation		
Name:	Side:	Left	Right
Injury date:	Operation date:		

Pain (tick one box in each column)		
	Before Injury	Current
None		
Mild—no limit to activity		
Moderate—limit of strenuous exercise		
Fairly severe—some limitation of day‐to‐day activity		
Very severe—severe limitation		
Totally disabled		

Running/walking (circle one in each column)	
Before injury	Current
Sprint/Run/Jog/Brisk Walk/Walk/Unable to walk	Sprint/Run/Jog/Brisk Walk/Walk/Unable to walk

Activity (tick one box in each column)		
	Before injury	Current
Unrestricted		
Mildly restricted		
Unable to do strenuous exercise		
Unable to do moderate exercise		
Unable to do gentle exercise		
Unable to do activities of daily living		

Sitting/driving (tick one box in each column)		
	Before injury	Current
Any type of seat as long as you like		
Comfortable seat for as long as you like		
Pain stops me driving/sitting for more than an hour		
Pain stops me driving/sitting for more than 30 min		
Pain stops me driving/sitting for more than 10 min		
Unable to sit/drive		

Stairs/inclines		
	Before injury	Current
Easily		
Little difficult		
Moderate difficulty (stop frequently)		
Extreme difficulty (use of aids intermittently)		
Only with support of aids (every step)		
Unable to do		

Aches and tightness (tick one box)				
	Before injury ache	Current ache	Before injury tightness	Current tightness
Never				
Only with exercise				
Rare (once a month)				
Occasional (1 week)				
Regularly (everyday)				
Always (constant)				

*Note*: The SHORE score is a patient‐reported outcome measure assessing pain, function and activity limitation before injury and at current follow‐up in patients with proximal hamstring injury.

### MRI

Analysis of MRI imaging was performed by a single musculoskeletal fellowship‐trained radiologist (S.F.). Assessment of muscle changes was based on qualitative assessment of signal intensity of the hamstring muscles, both in terms of the degree of signal intensity on fat‐saturated images as well as the percentage of cross‐sectional area involvement of the muscle on axial scans. Oedema and muscle atrophy were assessed and graded according to intensity (none, mild = 1%–33%, moderate = 34%–66%, severe > 67%–100%), consistent with established MRI descriptions of denervated skeletal muscle. Acute or subacute denervation‐related changes were identified by increased T2/short tau inversion recovery (STIR) signal intensity, while chronic changes were characterised by muscle atrophy and fatty infiltration, as described in prior experimental and clinical studies of peripheral nerve injury [12, 29].

Oedema was considered reticular or linear increased soft tissue signal, scar tissue was considered linear or band‐like low signal in the soft tissues, both which were determined to involve the epineurium of the sciatic nerve if there was no clear fat plane between it and the surface of the nerve. Haematoma was considered to be fluid signal on T2 imaging or high signal on T1 imaging in the acute setting. This grading was performed for all four muscles of the proximal hamstring (semimembranosus, semitendinosus, short and long head of biceps femoris), thus giving each a numerical grading between 0 and 9, ascending with pathological change. The sum of all gradings was deemed the MRI total score.

Although MRI‐based classification systems for proximal hamstring tears and avulsions have been described, no validated MRI scoring system currently exists for quantifying denervation‐related muscle changes or perineural soft‐tissue involvement in correlation with EMG‐confirmed neurotrauma [6]. Accordingly, this ordinal grading approach was defined a priori to provide a clinically interpretable summary of conventional MRI findings relevant to neural risk. Formal intra or interobserver reliability analysis was not performed.

### Neurophysiology

Nerve conduction studies (K.N.) consisted of the measurement of sensory and motor amplitudes with conduction velocities from sural and superficial peroneal nerves, and from extensor digitorum brevis and abductor hallucis muscles. Long latency F‐wave recordings were also recorded from motor studies. Needle EMG was routinely performed in the following muscles: tibialis anterior, medial gastrocnemius and the four hamstring muscles of semitendinosus, semimembranosus, long and short head of biceps. The presence of acute electrophysiological denervation changes was noted, and a binary outcome of normal or abnormal was assigned if there were any features of denervation. This could consist of a high‐firing motor unit pattern on needle EMG, especially if intervention was within a week, ± the presence of spontaneous activity if the injury was at least a week old.

An abnormal EMG was defined as the presence of either spontaneous activity on needle EMG, which was more commonly observed or neurogenic motor unit changes, which were less common in the early phase. Spontaneous activity included fibrillation potentials and positive sharp waves, while neurogenic changes were characterised by larger‐amplitude, prolonged‐duration or polyphasic motor unit potentials. A binary outcome of normal or abnormal EMG was assigned if any of these features were present.

Muscles were sampled at a site distal to any MRI changes, to minimise confounding traumatic changes for spontaneous activity. In this way, sciatic trunk injuries could be assessed from nerve conduction and needle EMG of the muscles below the knee, and branch injuries after leaving the trunk proper by needle EMG of the hamstring muscles, enabling early detection of denervation or conduction block from injury.

### Surgical procedure

All surgical procedures were performed by the senior author (D.W.). Surgery was performed in a standardised, previously published technique [7]. In all cases, three suture anchors were utilised for repair and fixation (Q‐Fix All‐Suture; Smith & Nephew), and routine sciatic nerve identification and neurolysis was performed.

### Postoperative study protocol

All participants were followed up at three months postoperatively. Follow‐up included a routine postoperative clinical consultation with the senior surgeon (D.W.), and an nerve conduction studies (NCS)/EMG and MRI at 3, 6 and 12 months (K.N.). Participants with abnormal EMG findings preoperatively and/or 3 months postoperatively were followed up again at 6 months postoperatively. A final follow‐up was conducted at 12 months postoperatively for those participants with abnormal EMG findings at 6 months. Two consecutive normal NCS/EMG results signifying either no injury at any point, or sufficient recovery after an abnormal result, warranted termination of further neurophysiological testing. Participants wishing to withdraw at any point were recorded and not contacted for further follow‐up.

### Statistical analysis

All statistical analyses were carried out using SPSS (Version 29, SPSS Inc.). Continuous variables were calculated as means and categorical variables as frequency counts. All continuous data were checked for the assumptions of linearity, normality, homoscedasticity, independence of observations and multicollinearity.

**Figure 1:**
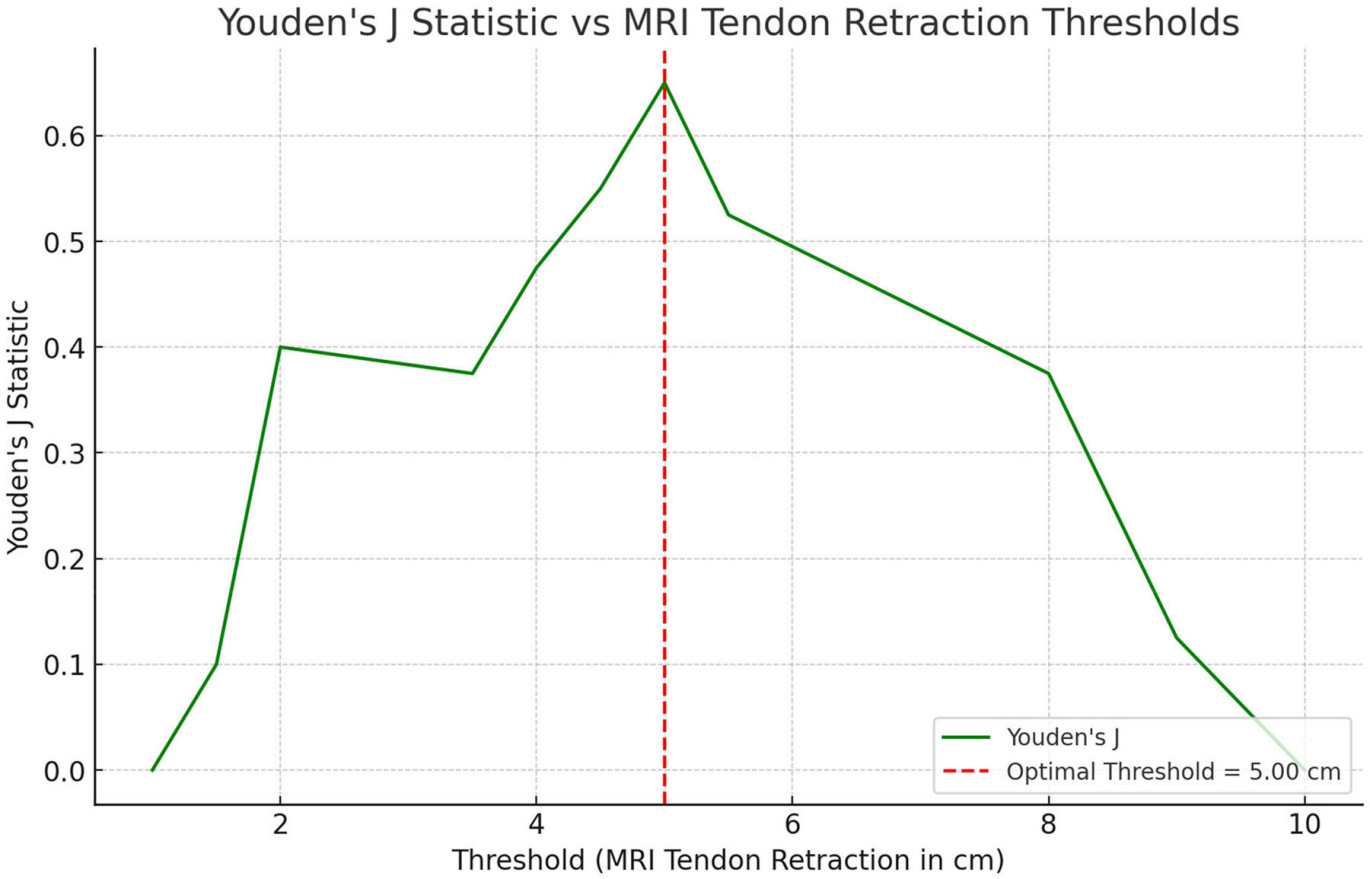
Statistic 1. Youden's *J*‐statistic plotted against increasing magnetic resonance imaging (MRI) tendon retraction distance thresholds (cm) for predicting abnormal electromyography (EMG) findings following proximal hamstring avulsion. The optimal threshold corresponds to the maximal Youden's *J*‐value.

**Figure 2:**
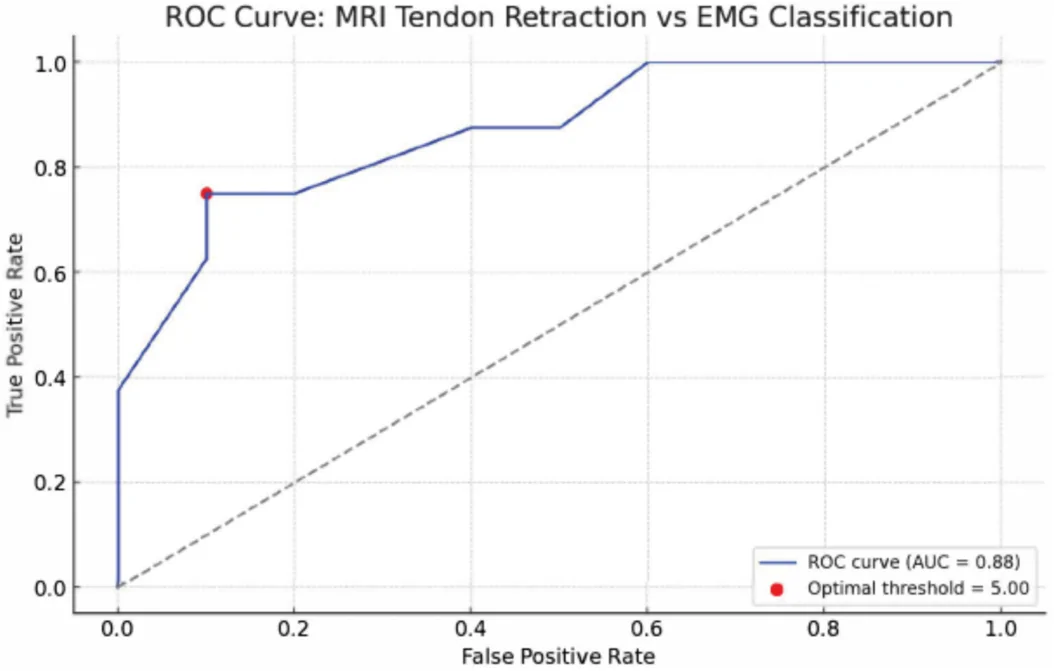
Receiver operating characteristic (ROC) curve demonstrating the diagnostic performance of magnetic resonance imaging (MRI)–measured proximal hamstring tendon retraction distance for predicting abnormal electromyography (EMG) findings following proximal hamstring avulsion. Sensitivity is plotted against 1–specificity across increasing retraction distance thresholds. The area under the curve (AUC) was 0.88, indicating good discriminatory ability. The optimal cutoff value was determined using Youden's J statistic, with a threshold of 5.0 cm corresponding to a sensitivity of 0.75 and specificity of 0.90. *n* = 18.

Between‐group differences for baseline demographic, clinical and surgical data were analysed using independent *t*‐tests for continuous data or chi‐square test for independent dichotomous data. The ability of MRI‐measured tendon retraction distance to discriminate with normal or abnormal EMG findings was assessed using receiver operating characteristics (ROC) curve analysis. Tendon retraction distance was treated as a continuous predictor and EMG findings were dichotomised as normal or abnormal. Discriminatory performance was quantified using the ROC curve (area under the curve [AUC]) with corresponding confidence intervals. The optimal threshold was determined using Youden's *J*‐statistic (sensitivity + specificity – 1), with sensitivity and specificity reported for the selected cut‐off. An AUC of 0.5 indicated no discrimination. Figures 1 and 2; Statistics 1 and 2).

No a priori power or sample size calculation was performed, as this study was exploratory and hypothesis‐generating.

## RESULTS

### Etiology and trajectory of nerve injury

**Figure 3:**
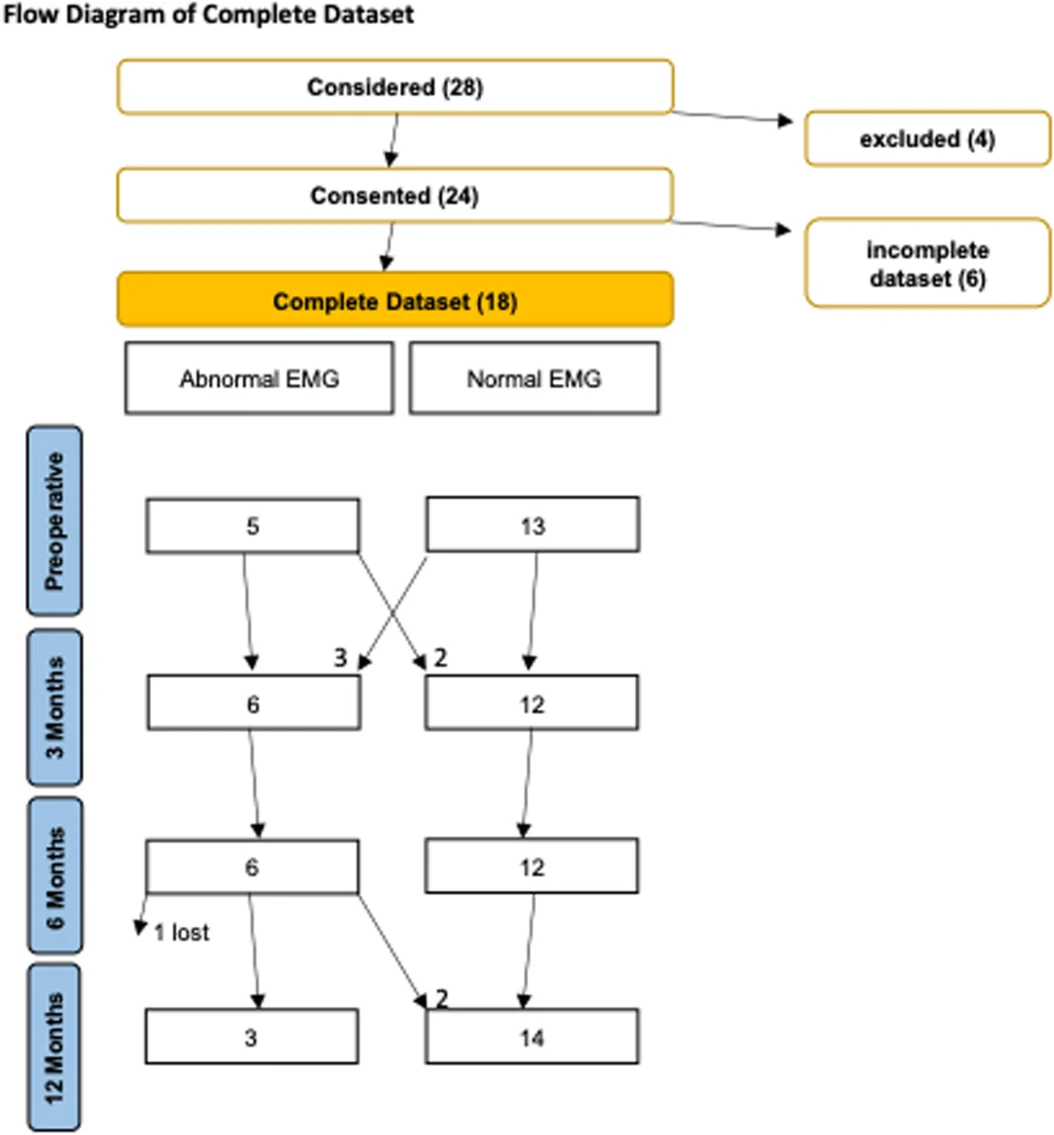
CONSORT flow diagram illustrating patient identification, eligibility assessment, exclusions and final cohort inclusion for analysis of proximal hamstring avulsion injuries, including allocation to normal and abnormal electromyography (EMG) groups for subsequent radiological and clinical analyses.

Of 28 eligible participants who underwent surgical repair of their proximal hamstring avulsion, 24 met inclusion criteria and agreed to participation between September 2020 and July 2021. Six participants were excluded due to incomplete preoperative EMG data. (Figure 3; Consort Flow Diagram). Eighteen participants were included in the final analysis (15 males, 3 females), with a mean age of 54 years (SD 11.3). Injury laterality, mechanism of injury, injury classification and preinjury activity level are summarised in Table 2. Preoperatively, 5 participants had abnormal EMG studies. By 3 months, 2 abnormal EMG studies normalised, while 3 additional participants developed abnormalities. At 12 months, 2 additional abnormal EMGs normalised, resulting in 14 normal EMG results, 3 abnormal and 1 lost to follow‐up.

**Table 2:** Comparison of normal and abnormal EMG cohorts of patients with proximal hamstring avulsion.

Variable	Total cohort (*n* = 18)	EMG classification subgroup		*p*‐value
		Normal (*n* = 10)	Abnormal (*n* = 8)	
	Mean (SD)	Mean (SD)	Mean (SD)	
Age (years)	54 (11.2)	54 (10.9)	53 (11.5)	0.83
Time from injury to surgery (days)	26 (15.5)	27.9 (19.7)	23.6 (6.7)	0.59
Retraction distance (cm)	4.3 (2.4)	2.9 (1.5)	5.9 (2.2)	0.005*
SHORE scores				
Preinjury	32.94 (1.72)	33.4 (1.3)	32.4 (2.2)	0.23
Preoperative	7.17 (4.67)	6.3 (3.8)	8.3 (5.9)	0.41
6‐months	29.39 (2.69)	29.6 (2.3)	29.1 (3.4)	0.73
12‐months	31.61 (1.57)	31.8 (1.0)	31.4 (2.2)	0.60
	***n* (%)**	***n* (%)**	***n* (%)**	
Sex				0.81
Male	13 (72.2)	7 (70)	6 (75)	
Female	5 (31.8)	3 (30)	2 (25)	
Side of injury				0.91
Right	11 (61.1)	6 (60)	5 (62.5)	
Left	7 (42.3)	4 (40)	3 (37.5)	
Injury mechanism				0.87
Stretch	16 (88.8)	9 (90)	7 (87.5)	
High speed running/sprint	2 (11.12)	1 (10)	1 (12.5)	
Injury mechanism velocity				0.53
Low	6 (33.3)	3 (30)	3 (37.5)	
Medium	7 (38.9)	5 (50)	2 (25)	
High	5 (30.0)	2 (20)	3 (37.5)	
Previous treatment				0.25
Yes	1	0	1	
No	17	10	7 (12.5)	
Injury classification				0.50
V	6 (33.3)	4 (40)	2 (25)	
VI	12 (66.7)	6 (60)	6 (75)	
Preoperative sciatic nerve appearance (MRI)				0.91
Normal	7 (38.9)	4 (40)	3 (37.5)	
Abnormal	11 (61.1)	6 (60)	5 (62.5)	
Presence of preoperative neurological symptoms				0.07
Yes	7 (38.9)	2 (20)	5 (62.5)	
No	11 (61.1)	8 (80)	3 (37.5)	
Presentation of injury				0.18
Acute	16 (0.9)	8 (80)	8 (100)	
Chronic	2 (0.1)	2 (20)	0	
Previous activity level				0.63
Elite	1	1 (10)	0 (0)	
Competitive	7	4 (40)	3 (27.5)	
Recreational	10	5 (50)	5 (62.5)	
Postoperative complication				0.20
Yes	6	4	1	
DVT		1	0	
Hamstring muscle atrophy		1	0	
Reduced ROM		1	0	
Sitting pain		1	0	
New numbness		1	1	
No	11	6	7	

*Note*: Demographic, clinical, imaging and functional variables are compared between patients with normal and abnormal EMG findings. Continuous variables are reported as mean (± standard deviation) and categorical variables as number (percentage). *p*‐Values refer to between‐group comparisons. Asterisk (*) Statistical significance was defined as *p* < 0.05.

Abbreviations: DVT, deep vein thrombosis; EMG, electromyography; MRI, magnetic resonance imaging; ROM, range of motion; SD, standard deviation.

### Injury outcomes

SHORE scores did not significantly differ between normal and abnormal EMG cohorts at any time‐point (Table 1). There were no other significant differences between those with normal and abnormal EMG, in various characteristics, including the mechanism of injury, such as the velocity, the injury classification or the previous activity level of the subjects.

Overall, there were no statistically significant differences between total MRI Scores and EMG results (Tables 2 and 3). There were some differences in the MRI appearance of the muscles in denervated versus normal muscles defined by EMG at some time points (e.g., preoperatively for the semimembranosus muscle), but overall, there was not a strong correlation. No difference in MRI appearances of the sciatic nerve between these groups dichotomised by neurophysiology.

**Table 3:** Comparison of muscle MR composite scores by normal and abnormal EMG result.

	Preoperative		*p*‐ value	3‐month post‐op		*p*‐ value	6‐month post‐op		*p*‐ value	12‐months post‐op		*p*‐ value
	Normal	Abnormal		Normal	Abnormal		Normal	Abnormal		Normal	Abnormal	
	Mean (SD)	Mean (SD)		Mean (SD)	Mean (SD)		Mean (SD)	Mean (SD)		Mean (SD)	Mean (SD)	
SH	0.5 (0.89)	0.5 (0.71)	1.00	0.0 (0.00)	0.0 (0.00)	‐	0.00 (0.00)	0.00 (0.00)	‐	0.25 (0.50)	2.00 (0.00)	0.00*
	*n* = 16	*n* = 2		*n* = 15	*n* = 3		*n* = 4	*n* = 4		*n* = 4	*n* = 3	
LH	1.38 (1.26)	1.50 (0.71)	0.89	3.50 (2.03)	5.50 (2.65)	0.12	5.25 (2.06)	5.00 (2.45)	0.88	5.20 (2.17)	8.00 (1.41)	0.16
	*n* = 16	*n* = 2		*n* = 14	*n* = 4		*n* = 4	*n* = 4		*n* = 5		
SM	1.44 (0.89)	3.50 (3.54)	0.04*	2.85 (2.27)	5.00 (2.65)	0.10	4.00 (2.83)	4.33 (2.42)	0.86	4.25 (2.06)	7.33 (3.06)	0.17
	*n* = 16	*n* = 2		*n* = 13	*n* = 5		*n* = 2	*n* = 6		*n* = 4	*n* = 3	
ST	0.93 (0.96)	1.33 (0.58)	0.50	2.67 (1.88)	5.00 (2.00)	0.07	2.75 (1.50)	4.75 (1.26)	0.09	2.75 (1.50)	6.33 (1.52)	0.03*
	*n* = 15	*n* = 3		*n* = 15	*n* = 3		*n* = 4	*n* = 4		*n* = 4	*n* = 3	

*Note*: Mean (± standard deviation) MRI composite scores are shown for individual hamstring muscles preoperatively and at 3, 6 and 12 months postoperatively. Values are reported separately for patients with normal and abnormal EMG findings at each time point. *n* denotes the number of patients available for analysis. *p*‐Values refer to between‐group comparisons. Asterisk (*) Statistical significance was defined as *p* < 0.05. A hyphen (–) indicates that comparative analysis was not performed.

Abbreviations: EMG, electromyography; MR, magnetic resonance; SD, standard deviation; SH, short head of biceps femoris; SM, semimembranosus; ST, semitendinosus.

### Preoperative tendon retraction distance and nerve injury

When the abnormal EMG cohort was compared with the normal EMG cohort, a significant difference between the cohorts was only observed in preoperative tendon retraction distance (Table 2). While more participants in the abnormal EMG cohort reported neurological sensory symptoms preoperatively, the difference was not statistically significant between cohorts (*p* = 0.07; Table 3).

#### Tendon retraction distance versus EMG

There was a significant difference in preoperative tendon retraction distances between the normal and abnormal EMG cohorts (5.9 cm vs. 2.9 cm; *p* = 0.005). Calculated Cohen's *d* (1.59) CI (0.657, 2.516), These findings indicate a significant large effect size, suggesting a substantial difference in tendon retraction distance between the normal and abnormal EMG groups. The ROC AUC was 0.88, indicating excellent discrimination ability. (Figure 1, Youdens *J*‐Statistic 1). Sensitivity of 90% (95% CI: 66.7%–100%), and specificity of 75% (95% CI: 40%–100%). Cross‐validation confirmed a mean AUC of 0.80 (SD: 0.19).

Optimal threshold analysis based on Youden's *J*‐statistic was 5 cm. (Figure 2, ROC Tendon Retraction Distance, Statistic 2.).

## DISCUSSION

This study demonstrates that proximal hamstring avulsion injuries are frequently associated with occult neurotrauma, which may be traumatic or iatrogenic in origin. EMG‐confirmed denervation was present preoperatively in a substantial proportion of patients and developed postoperatively in a further subset. Greater MRI‐measured tendon retraction distance was strongly associated with the presence and severity of neurotrauma, whereas conventional MRI muscle signal changes did not reliably correlate with functional denervation.

Surgical repair of acute complete proximal hamstring ruptures is generally associated with high patient satisfaction and favourable return‐to‐sport outcomes [4, 5, 17]. This is supported by systematic reviews of operative management [5, 10].

Nevertheless, delayed recovery and persistent hamstring weakness may occur in a subset of patients, suggesting a neuropathological contribution [3, 27]. Sciatic nerve–related symptoms have been reported in approximately one quarter of patients, although motor and sensory deficits are less common and recovery appears more favourable following operative treatment [8, 21, 30].

### Anatomy and innervation

**Figure 4:**
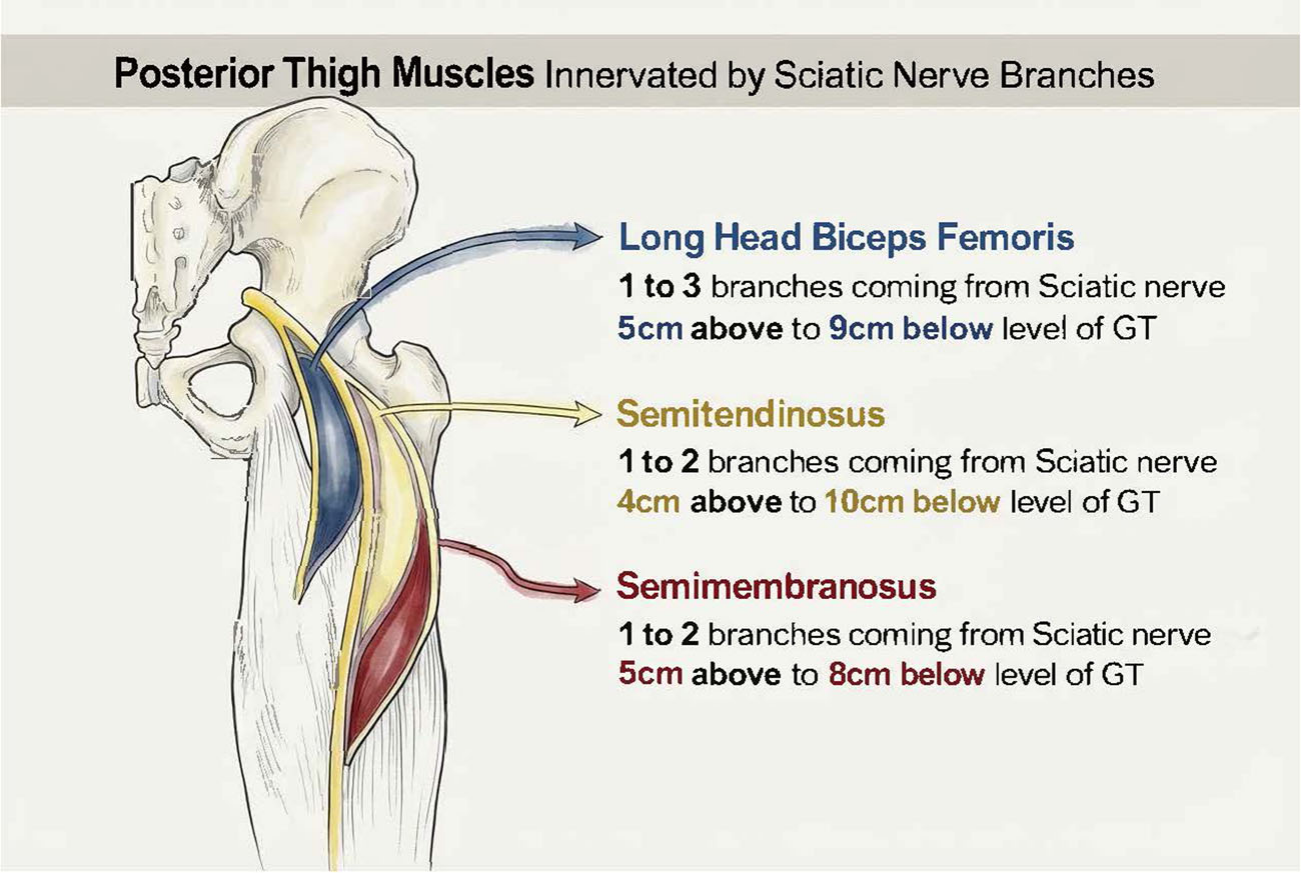
Innervation proximal hamstring. Schematic illustration of proximal hamstring innervation showing the branching patterns of the tibial division of the sciatic nerve to the long head of biceps femoris, semitendinosus and semimembranosus muscles. Coloured areas indicate variability in motor branch anatomy.

The anatomical proximity of the sciatic nerve to the ischial tuberosity renders it vulnerable to injury both at the time of avulsion and during surgical repair [13, 19, 21, 26]. Seidel et al. described distinct zones of motor branch distribution, highlighting a complex branching pattern rather than dominance by a single motor branch. (Figure 4, innervation of the proximal hamstring.) In the present study, greater tendon retraction was associated with a higher likelihood of EMG abnormalities both pre and postoperatively. A nerve‐at‐risk distance (NARD) of 5 cm lies within Seidel's zone 1, which contains early motor branches to the hamstrings [22]. Prior anatomical work has demonstrated that recurrent motor branches may lie as close as 1.5 cm from the ischial tuberosity, supporting the concept that increasing retraction distance represents an anatomical risk factor for neurotrauma [20, 22]. Tendon retraction distance should therefore be interpreted as a marker of neural risk rather than a direct causal determinant, as injury severity, mechanism and anatomical variability limit causal inference.

Longitudinal analysis demonstrated that all hamstring motor branches were susceptible to traumatic injury, with the branch to the semimembranosus muscle appearing most susceptible to iatrogenic injury. Delayed postoperative denervation was observed between 3 and 6 months in all muscles except the long head of biceps femoris. Among affected muscles, semimembranosus demonstrated the highest rate of recovery, with denervation persisting at 12 months in only half of cases identified at 6 months. This recovery pattern may reflect anatomical or surgical factors, although the present data are insufficient to confirm a mechanism.

In addition to acute or iatrogenic injury, chronic tethering or compression of the sciatic nerve by scarred proximal hamstring tissue has been described as a cause of delayed neurologic symptoms, sometimes requiring surgical neurolysis [3, 19, 21]. Such presentations include proximal hamstring syndrome and extraspinal sciatica, which may manifest months or years after the index injury [18, 30]. Magnetic resonance neurography has demonstrated perineural scarring and nerve signal change in cases of chronic sciatic neuropathy related to remote proximal hamstring injury [6].

### MRI/EMG

In this cohort, MRI muscle signal abnormalities did not significantly correlate with EMG‐confirmed denervation, indicating that structural MRI alone is unreliable for predicting sciatic nerve injury. Acute muscle denervation has been shown to correlate with T2 hyperintensity and STIR signal changes in experimental and selected clinical settings [12, 25, 29]. In proximal hamstring avulsion, soft‐tissue trauma, extensive hematoma and inflammation may obscure denervation‐related changes on conventional sequences [2, 12].

Furthermore, clinically relevant nerve injury—particularly partial or early axonal injury—may occur without detectable MRI abnormalities on conventional sequences. MRI findings should therefore be interpreted cautiously and in conjunction with clinical assessment and EMG, which remains necessary to confirm functional denervation.

Advanced magnetic resonance neurography has shown promise in characterising sciatic neuropathy by directly depicting nerve signal change and perineural scarring, but remains less widely available and was not used in this cohort. Accordingly, the present findings apply specifically to conventional MRI protocols [6, 16].

### Limitations

This study was limited by a small sample size and a single‐surgeon cohort, introducing potential selection bias and limiting power to detect more subtle associations. The absence of a nonoperative comparison group precludes assessment of whether surgical repair reduces delayed noniatrogenic neural injury. MRI assessments were performed by a single musculoskeletal radiologist and EMG studies were interpreted by two neurologists without formal interobserver reliability analysis, which may limit reproducibility.

### Implications for treatment and management

MRI‐measured tendon retraction may aid prognostic stratification, while EMG remains essential for confirming functional denervation. A retraction threshold of 5 cm may identify patients at increased neurological risk. Future studies should evaluate surgical timing, technique and adjunct imaging modalities, including neuromuscular ultrasound, in prospective cohorts [23].

## CONCLUSION

Proximal hamstring avulsion injuries are associated with variable neuropathology and recovery. MRI‐measured tendon retraction is useful for defining nerve‐at‐risk distance but does not reliably identify denervation. EMG remains essential for confirming functional neurotrauma and characterising its pattern. A nerve‐at‐risk distance greater than 5 cm is associated with a substantially increased risk of neurotrauma, although the long‐term clinical significance of underlying nerve injury, particularly in athletes, requires further investigation.

## AUTHOR CONTRIBUTIONS

**David Wood**: Conceptualisation; methodology; surgical data acquisition; study design; data curation; investigation; writing—review and editing. **Milos Spasojevic**: Formal analysis; writing—original draft; writing—review and editing; project administration. **Sofie French**: Study design; data curation; investigation; formal analysis. **Ran Wei**: Data curation; data management; visualisation. **Sebastian Fung**: Radiological analysis; methodology; data acquisition. **Karl Ng**: Conceptualisation; electrophysiology expertise; methodology; validation; data interpretation; writing—review and editing; supervision; writing—review and editing.

## CONFLICT OF INTEREST STATEMENT

The authors confirm there are no conflicts of interest to disclose. The authors, their immediate family, and any research foundation with which they are affiliated did not receive any financial payments or other benefits from any commercial entity related to the subject of this article.

## ETHICS STATEMENT

Ethics approval was granted by St Vincent's Hospital Human Research Ethics (2019/ETH10683).

## Data Availability

Data available on request due to privacy/ethical restrictions. The data that support the findings of this study are available on request from the corresponding author. The data are not publicly available due to privacy or ethical restrictions.
